# Understanding Discontinuation Rates and Acceptance of the Low-Dose Levonorgestrel Intrauterine System in Spain: A Comprehensive Analysis of Bleeding Patterns and Influencing Factors

**DOI:** 10.1089/whr.2024.0113

**Published:** 2025-03-05

**Authors:** Josep Perelló-Capó, Gregorio López-González, María Isabel Lahoz-Pascual, Ana Isabel López-Castejón, Manuel Marcos-Fernández, Mercedes Andeyro-García, Ignacio Cristóbal-García, Joan Rius-Tarruella

**Affiliations:** ^1^ Department of Paediatrics, Obstetrics and Gynaecology, Santa Creu i Sant Pau Universitary Hospital, Barcelona, Spain.; ^2^ Preventive Medicine and Public Health, Universitat Autònoma de Barcelona, Barcelona, Spain.; ^3^ Department of Obstetrics and Gynaecology, Hospital Universitario 12 de Octubre, Madrid, Spain.; ^4^ Department of Obstetrics and Gynaecology, Hospital Clínico Universitario Lozano Blesa, Zaragoza, Spain.; ^5^ Department of Obstetrics and Gynaecology, HM Hospitales, Madrid, Spain.; ^6^ Department of Obstetrics and Gynaecology, Hospital Universitario HM Montepríncipe, Madrid, Spain.; ^7^ Department of Obstetrics and Gynaecology, Hospital Universitario General de Villalba, Madrid, Spain.; ^8^ Department of Obstetrics and Gynaecology, Hospital Clínico San Carlos, Madrid, Spain.; ^9^ Medical Department, Medical Advisor Women Health, Bayer Hispania S.L., Barcelona, Spain.

**Keywords:** low-dose levonorgestrel-releasing intrauterine device, discontinuation rate, acceptability, satisfaction, menstrual bleeding pattern, real-life data

## Abstract

**Purpose:**

To estimate the 1-year continuation rate of low-dose levonorgestrel-releasing intrauterine systems (LNG-IUS) in Spanish women and elucidate potential factors impacting continuation.

**Materials and Methods:**

A prospective, multicenter, noninterventional study with a 1-year follow-up was conducted in Spain. Participants were 18–35-year-old women using low-dose LNG-IUS. Clinical and demographic data were collected, and the association between baseline characteristics and discontinuation rate was analyzed.

**Results:**

A total of 289 women (9.3% using 13.5 mg LNG-IUS and 90.6% using 19.5 mg LNG-IUS) completed the study, and 9% discontinued prematurely after 12 months. A statistically significant association was found between LNG-IUS discontinuation and educational level (odds ratio [OR] = 2.63; 95% confidence interval [CI]: 1.07–6.48), previous pregnancies (OR = 3.44; 95% CI: 1.40–8.46), and baseline intensity of menstrual pain (OR = 1.03; 95% CI: 1–1.04). In addition, both the change in the menstrual bleeding’s interference with daily life activities between the final and basal visit and the change in the pain associated with the intensity of menstrual bleeding showed a significant association with discontinuation.

**Conclusions:**

When recommending LNG-IUS, a patient’s baseline characteristics such as educational level, previous pregnancies, intensity of menstrual pain, and menstrual bleeding’s interference with daily life activities have to be considered. By doing so, health care providers can improve contraceptive counseling, reduce discontinuation rates, and enhance women’s satisfaction.

## Introduction

Long-acting reversible contraceptives (LARCs) are highly safe and effective methods recommended by international guidelines as the first contraceptive option.^1,2^ Mainly, there are two types of LARCs: intrauterine devices (IUD) and subdermal implants. Among the 966 million women of reproductive age using any method of contraception, only around 16.8% are using IUD,^3^ with rates ranging from 1% to 2% in Canada to 41% in China.^4^ In Spain, according to the data from the latest contraception survey by the Spanish Society of Contraception, 6.7% of women of reproductive age use an intrauterine method: 3.8% use a hormonal intrauterine system (levonorgestrel-releasing intrauterine system [LNG-IUS]), and 2.9% use a copper IUD (Cu-IUD).^5^ Both intrauterine methods are highly effective but differ in their mechanism of action. When reviewing the most used system, LNG-IUS, it has reported high efficacy together with low failure rates, between 0.1% and 0.2%, within the first year.^4^ This high effectivity translates into low rates of unintended pregnancy. Besides these clinical and personal benefits, LNG-IUS are also cost-effective despite high initiation costs.^6^

Noteworthy, most LNG-IUS users have reported irregular bleeding patterns due to the local effect of levonorgestrel on the endometrium. In particular, a dose-dependent reduction in the number of bleeding and/or spotting days and the number of bleeding episodes (amenorrhoea).^7^ Among high-dose LNG-IUS (52 mg) users, rates of amenorrhea and infrequent bleeding are higher than in low-dose LNG-IUS (13.5 mg and 19.5 mg) users.^8,9^ Although discontinuation rates due to bleeding changes are low in clinical trials (around 5%),^8^ multiple studies have reported that menstrual bleeding changes due to contraceptives are important reasons for contraceptive dissatisfaction and discontinuation, especially in the first year.^7,10^

Although bleeding pattern changes are a recognized cause of LNG-IUS discontinuation, few studies have evaluated the associated risk factors. Understanding the relationship between the postinsertion bleeding pattern and the continuity in low-dose LNG-IUS users would help health care professionals (HCPs) better comprehend their behavior regarding bleeding patterns and provide relevant information for improved contraceptive counseling. Therefore, the main objective of this study was to estimate the 1-year continuation rate of low-dose LNG-IUS in relation to the menstrual bleeding pattern in 18–35-year-old Spanish women. The secondary objectives of the study were to identify potential factors impacting the continuation rate of the low-dose LNG-IUS and to estimate the association of the discontinuation rate with changes in the bleeding pattern.

## Material and Methods

### Study design and participants

An observational, prospective, multicenter, noninterventional study was conducted to estimate the continuation rates and user acceptability of bleeding profiles in first-time users of LNG-IUS in Spain. Spanish centers, public and private, were consecutively recruited between 21 February 2021 and 20 December 2021.

Women aged between 18 and 35 years, who had chosen any of the marketed low-dose LNG-IUS in Spain for the first time in their lives (19.5 mg LNG-IUS or 13.5 mg LNG-IUS) during a routine gynecological visit after being adequately counseled and informed of all contraceptive options by their physician, were consecutively included. The insertion of the LNG-IUS was conducted during the baseline visit.

Women with the ability to read and write, sign the consent form, capable of making decisions and following instructions where low-dose is not contraindicated, and without concurrent medication that may alter the bleeding pattern.

Women were informed about the study and invited to participate. Written informed consent was obtained from every woman who voluntarily participated in the study.

### Data collection and analysis

Data collection was carried out in two or three visits: the baseline visit, the follow-up visit (optional), and the final visit (after approximately 12 months from the initial visit or at discontinuation with the LNG-IUS, if earlier). These visits took place in a routine clinical practice setting. Investigators collected demographic and clinical data from medical records and personal interviews. Data collection was carried out using a case report form (CRF).

Overall satisfaction with LNG-IUS was measured by a 5-point Likert scale: “very satisfied,” “satisfied,” “neither satisfied nor dissatisfied,” “dissatisfied,” and “very dissatisfied.” The quantity of menstrual bleeding was measured on a 10 cm visual analog scale (VAS) (on the left, “absence of bleeding”; on the right, “worst imaginable bleeding”). Menstrual pain was quantified on a 10 cm VAS (on the left, “no pain”; on the right, “worst imaginable pain”).

### Statistical methods

A descriptive analysis was performed for all variables. Continuous variables were characterized using mean and standard deviation (SD). For categorical variables, numbers (n) and frequencies (percentages) were calculated.

Continuous variables were analyzed using the Wilcoxon two-sample test. Categorical variables were analyzed using the Chi-squared test or Fisher’s exact test.

To study the factors in the regression data analysis, the odds ratio was calculated between the variables of interest and the discontinuation ratio, followed by logistic regression using the stepwise method. All applicable statistical tests were two-sided with a 5% significance level. The Wald test from logistic regression was used. Besides, a linear correlation between two variables was also performed. Also, Kaplan–Meier survival curves were used to describe time to discontinuation.

A sample size of 308 women has been calculated to be powered enough to estimate a discontinuation rate of up to 20% after 12 months with a margin error of 5%, two-sided confidence level of 95%, and assuming 20% loss of follow-up. A simple asymptotic approach without continuity correction has been used for the sample size estimation.

### Ethical considerations

The study was approved by the ethics committee of the Hospital de la Santa Creu i Sant Pau of Barcelona (EC/20/323/6113 OBS) authorized by the Agencia Española de Medicamentos y Productos Sanitarios (AEMPS).

## Results

**FIG. 1.**
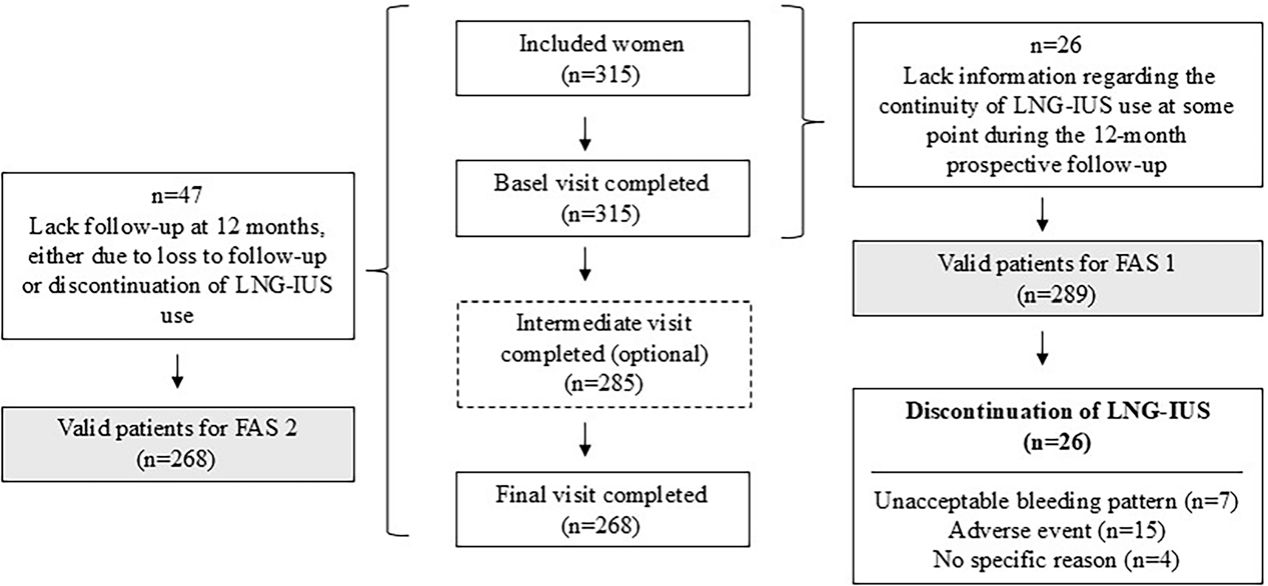
Flowchart of the study.

A total of 315 women were enrolled in the study. The basal visit was completed by all 315 women included in the study. An optional intermediate visit was completed by 285 women, and the final visit was completed by 268 women. Of the 315 women initially included, 26 were lost to follow-up during the 12-month prospective period, with no available information about the continuity of LNG-IUS use. A sample of 289 women was considered valid for the analysis of the primary objective and included in the full analysis set (FAS 1). FAS 2 included those women who started follow-up and completed them at 12 months (*n* = 268) (Fig. 1).

### Baseline demographic and clinical characteristics of the study population

Among 289 included women, 27 (9.3%) were using 13.5 mg LNG-IUS, and 262 (90.6%) were using 19.5 mg LNG-IUS. The number of subjects included was unbalanced between groups. Baseline demographic and clinical characteristics of participants according to the chosen device, 13.5 mg or 19.5 mg LNG-IUS, are shown in Table 1. Women using 13.5 mg LNG-IUS had a higher education, different working status (more students), and different marital status (more singles) than women included in the 19.5 mg LNG-IUS group. Significantly more women in the 19.5 mg LNG-IUS group reported a previous pregnancy (14.81% vs. 50%, *p* = 0.0005) (Table 1).

**Table 1.** Demographic and Clinical Characteristics of Included Women

Characteristic	13.5 mg LNG-IUS *n* = 27 (9.3%)	19.5 mg LNG-IUS *n* = 262 (90.6%)	Total *n* = 289 (100%)	*p* value
Demographics				
Age (years), mean (SD)	25.78 (4.41)	27.4 (4.93)	27.25 (4.9)	0.0853^a^
Place of birth, *n (%)*				0.0637^b^
Spain	24 (88.9%)	174 (66.4%)	198 (68.51%)	
Rest of Europe	0 (0.0%)	13 (5.0%)	13 (4.5%)	
South/Center America	2 (7.4%)	67 (25.6%)	69 (23.88%)	
North America	1 (3.7%)	1 (0.4%)	2 (0.69%)	
Asia	0 (0.0%)	5 (1.9%)	5 (1.73%)	
Africa	0 (0.0%)	2 (0.8%)	2 (0.69%)	
Level of studies, *n (%)*	** **	** **		**0.0011** ^b^
No studies/incomplete primary studies	0 (0.00%)	5 (1.91%)	5 (1.73%)	
Primary studies	0 (0.00%)	8 (3.05%)	8 (2.77%)	
Secondary studies	0 (0.00%)	33 (12.60%)	33 (11.42%)	
High school or tertiary	4 (14.81%)	106 (40.46%)	110 (38.06%)	
University studies	23 (85.19%)	110 (41.98%)	133 (46.02%)	
Working status^c^, *n (%)*	** **	** **	** **	**0.0457** ^b^ ** **
Active	17 (62.96%)	180 (69.50%)	197 (68.88%)	
Unemployed	0 (0.00%)	23 (8.88%)	23 (8.04%)	
Unpaid household work	0 (0.00%)	14 (5.41%)	14 (4.9%)	
Student	10 (37.04%)	41 (15.83%)	51 (17.83%)	
Pensioner or disability	0 (0.00%)	1 (0.39%)	1 (0.35%)	
Marital status, *n (%)*	** **	** **		**0.0078** ^b^
Married	3 (11.11%)	52 (19.85%)	55 (19.03%)	
Single	17 (62.96%)	71 (27.10%)	88 (30.45%)	
Stable partner	7 (25.93%)	132 (50.38%)	139 (48.1%)	
Divorced	0 (0.00%)	6 (2.29%)	6 (2.08%)	
Other	0 (0.00%)	1 (0.38%)	1 (0.35%)	
Gynecological				
Use of contraceptive method previous to IUD, *n (%)*				1
No	2 (7.41%)	25 (9.54%)	27 (9.34%)	
Yes	25 (92.59%)	237 (90.46%)	262 (90.66%)	
Time of use of the contraceptive method (if affirmative)	*n* = 25	*n* = 237	*n* = 262	**0.0176** ^b^
Less than 6 months	1 (4.00%)	48 (20.25%)	49 (18.7%)	
6 months to 1 year	1 (4.00%)	23 (9.70%)	24 (9.16%)	
1 to 3 years	12 (48.00%)	51 (21.52%)	63 (24.05%)	
More than 3 years	11 (44.00%)	115 (48.52%)	126 (48.09%)	
Previous pregnancies				**0.0005** ^d^
Yes	4 (14.81%)	131 (50.00%)	135 (46.71%)	
No	23 (85.19%)	131 (50.00%)	154 (53.29%)	
History of abortions	1 (25%)	70 (53.44%)	71 (52.59%)	0.345^b^
1	1 (25.00%)	54 (41.22%)	55 (40.74%)	
2	0 (0%)	11 (8.40%)	11 (8.15%)	
>3	0 (0%)	5 (3.82%)	5 (3.7%)	
Desire to have more children (if previous pregnancy)	*N* = 4	*N* = 131	*N* = 135	
Yes	3 (75.00%)	43 (32.82%)	46 (34.07%)	0.3184^b ^
No	1 (25.00%)	63 (48.09%)	64 (47.41%)	
Unknown	0 (0.00%)	25 (19.08%)	25 (18.52%)	
Desire to have more children (if no previous pregnancy)	*N* = 23	*N* = 131	*N* = 154	
Yes	20 (86.96%)	89 (67.94%)	109 (70.78%)	0.1507^b^
No	2 (8.70%)	16 (12.21%)	18 (11.69%)	
Unknown	1 (4.35%)	26 (19.85%)	27 (17.53%)	

*p* Values in bold indicate statistical significance.

^a^ Wilcoxon two sample test.

^b^ Fisher’s exact test.

^c^ There are three missing values.

^d^ Chi-squared test.

IUD, intrauterine device; SD, standard deviation.

Regarding menstrual bleeding pattern at baseline, women using the 13.5 mg LNG-IUS device presented a greater quantity of menstrual bleeding (*p* = 0.0011) and more menstrual pain at baseline (*p* = 0.0006). Conversely, women using 19.5 mg LNG-IUS reported significantly more interferences of menstrual bleeding with daily life activities at baseline, compared with 13.5 mg LNG-IUS users, who reported less frequently that it did not interfere “not at all” with their daily life (*p* = 0.0362). No differences regarding the frequency, regularity, duration of menstrual bleeding, and intermenstrual bleeding or spotting were observed between groups (Table 2).

**Table 2.** Characteristics of Menstrual Bleeding Pattern at Baseline According to Chosen Device

	13.5 mg LNG-IUS *n* = 27	19.5 mg LNG-IUS *n* = 262	Total *n* = 289	*p* value
Presence of menstrual bleeding				0.3768^a^
Yes	**27 (100.0%)**	**248 (94.66%)**	**275 (95.16%)**	
No	0 (0.00%)	14 (5.34%)	14 (4.84%)	
Intensity of menstrual pain, mean (SD)	52.93 (17.2)	34.68 (25.96)	36.58 (25.78)	**0.0006** ^b^
Quantity of menstrual bleeding (mm), mean (SD)	55.48 (14.17)	40.98 (21.75)	42.41 (21.54)	**0.0011** ^b^
Frequency of menstrual bleeding	*n* = 27	*n* = 248		0.8027^a^
Frequent (<24 days)	3 (11.11%)	41 (16.53%)	44 (16%)	
Normal (24–38 days)	23 (85.19%)	191 (77.02%)	214 (77.82%)	
Infrequent (>38 days)	1 (3.70%)	16 (6.45%)	17 (6.18%)	
Regularity of menstrual bleeding	*n* = 27	*n* = 248		0.0607^c^
Regular	24 (88.89%)	179 (72.18%)	203 (73.82%)	
Irregular	3 (11.11%)	69 (27.82%)	72 (26.18%)	
Duration of menstrual bleeding	*n* = 27	*n* = 248		0.9515^a^
Extended (>8 days)	4 (14.81%)	42 (16.94%)	46 (16.73%)	
Normal (4 to 8 days)	19 (70.37%)	173 (69.76%)	192 (69.82%)	
Short (<4 days)	4 (14.81%)	33 (13.31%)	37 (13.45%)	
Presence of intermenstrual bleeding or spotting	*n* = 27	*n* = 248		0.1343^c^
Yes	3 (11.11%)	59 (23.79%)	62 (22.55%)	
No	24 (88.89%)	189 (76.21%)	213 (77.45%)	
Menstrual bleeding interference with daily life activities	*n* = 27	*n* = 248		**0.0362**^a^
Not at all	13 (48.15%)	109 (43.95%)	122 (44.36%)	
Mild	10 (37.04%)	47 (18.95%)	57 (20.73%)	
Moderate	4 (14.81%)	61 (24.60%)	65 (23.64%)	
Intense	0 (0.00%)	31 (12.50%)	31 (11.27%)	

*p* Values in bold indicate statistical significance. *Data collection is carried out through a case report form (CRF)*.

^a^ Fisher’s exact test.

^b^ Wilcoxon two sample test.

^c^ Chi-squared test.

### Discontinuation rate

**FIG. 2.**
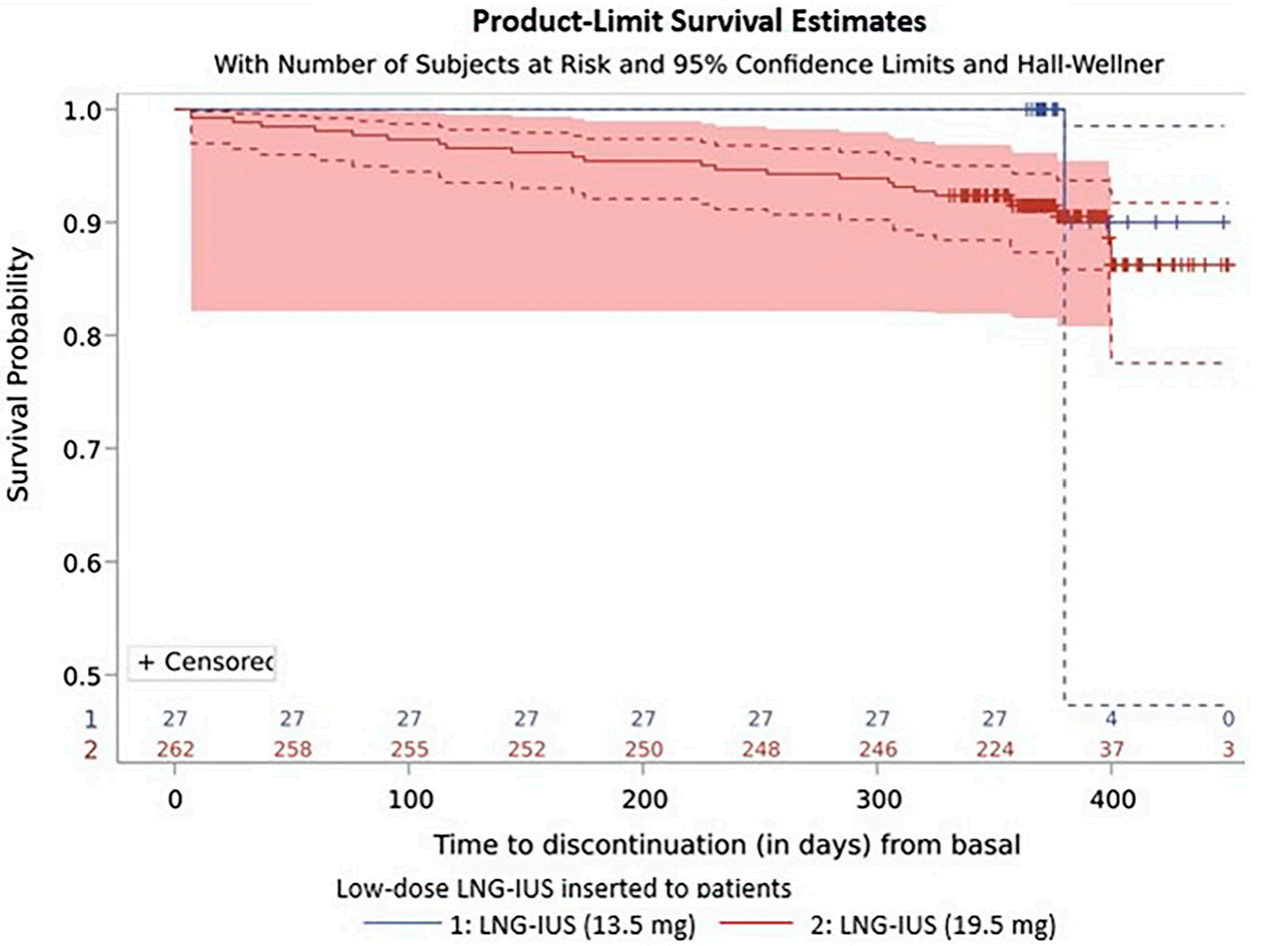
Time to discontinuation of participants depending on the chosen LNG-IUS. LNG-IUS, levonorgestrel-releasing intrauterine system.

In the 12-month observation period, premature discontinuation of low-dose LNG-IUS was observed in 26 women (9% of participants). Overall, reported reasons for discontinuation included presence of an unacceptable menstrual bleeding pattern (2.4%), adverse effects (5.2%), or other unspecified reasons (1.4%) (Table 3). Only one (3.7%) patient in the 13.5 mg LNG-IUS group discontinued, reporting unspecified reasons. In contrast, 9.5% of women discontinued in the 19.5 mg LNG-IUS group (Table 3). The mean time to discontinuation was 213 days (SD ± 134.07 days) (Fig. 2).

**Table 3.** Reasons for Discontinuation

	13.5 mg LNG-IUS *n* = 27	19.5 mg LNG-IUS *n* = 262	Total *n* = 289
Proportion of patients who discontinued, *n (%)*	1 (3.7%)	25 (9.5%)	26 (9.0%)
Reason for discontinuation	*n* = 1	*n* = 25	*n* = 26
Unacceptable menstrual bleeding pattern	0 (0.0%)	7 (28.0%)	7 (2.4%)
Adverse effects	0 (0.0%)	15 (60%)	15 (5.2%)
	—	*n* = 15	*n* = 15
Pelvic pain	—	7 (46.7%)	7 (46.7%)
LNG-IUS expulsion	—	3 (20.0%)	3 (20.0%)
Weight gain	—	1 (6.7%)	1 (6.7%)
Pregnancy (lack of effectiveness)	—	1 (6.7%)	1 (6.7%)
Acne	—	1 (6.7%)	1 (6.7%)
Abdominal bloating	—	1 (6.7%)	1 (6.7%)
Assorted AEs, such as weight gain, pelvic pain, and frequent spotting	—	1 (6.7%)	1 (6.7%)
No reason	1 (100%)	3 (12%)	4 (1.4%)

### Variables influencing discontinuation rate

To identify potential factors contributing to low-dose LNG-IUS discontinuation, a logistic regression analysis was performed. Table 4 shows the results of the logistic regression analysis. A statistically significant association was found between the following baseline characteristics and low-dose LNG-IUS discontinuation: educational level (OR = 2.63; 95% CI: 1.07–6.48), previous pregnancies (OR = 3.44; 95% CI: 1.40–8.46), and baseline intensity of menstrual pain (OR = 1.03; 95% CI: 1–1.04). When considering the change in recorded variables between the final visit and the basal visit, the menstrual bleeding’s interference with daily life activities showed a significant association with low-dose LNG-IUS discontinuation (OR = 3.89; 95% CI: 1.47–10.33) (Table 4).

**Table 4.** Possible Factors That May Contribute to Low-Dose LNG-IUS Discontinuation, (*n* = 289)

Factor	OR (95%CI)^a^	*p* value^a^
Age	0.99 (0.91–1.07)	0.985
Level of studies		
No studies (incomplete primary studies)/primary/secondary	2.63 (1.07–6.48)	**0.0353**
Secondary/University	Ref	
Working status		
Active	1.25 (0.27–5.73)	0.5312
Student/pensioner or disability	0.42 (0.06–3.18)	
Unpaid workhouse	0.81 (0.07–9.82)	
Unemployed	Ref	
Marital status		
Married/Stable partner	3.32 (0.32–34.09)	0.3834
Divorced/Widow	1.90 (0.69–5.23)	
Single	ref	
Use of contraceptive method previous to IUD		
Yes	2.74 (0.36–21.05)	0.3324
No	ref	
Time of use of the contraceptive method (if affirmative)		
Less than 1 year	1.84 (0.73–4.66)	0.3717
1 to 3 years	1.00 (0.33–3.06)	
More than 3 years	ref	
Type of IUD		
19.5 mg LNG-IUS	ref	0.3324
13.5 mg LNG-IUS	0.37 (0.05–2.80)	
Previous pregnancies		
Yes	3.44 (1.40–8.46)	**0.0071**
No	ref	
Presence of menstrual bleeding		
No (*n* = 289)	1.74 (0.37–8.25)	0.4835
Yes (*n* = 275)	ref	
Intensity of menstrual pain	1.03 (1–1.04)	**0.0037**
Quantity of menstrual bleeding	1.01 (0.99–1.03)	0.1984
Frequency of menstrual bleeding		
Frequent (<24 days)	Quasi-complete separation of data points detected.	
Normal (24–38 days)		
Infrequent (>38 days)		
Regularity of menstrual bleeding		
Irregular	0.72 (026–2.01)	0.5345
Regular	ref	
Duration of menstrual bleeding		
Short (<4 days)	7.03 (0.78–63.09)	0.2181
Normal (4 to 8 days)	4.66 (0.61–5.8)	
Extended (>8 days)	ref	
Presence/absence of intermenstrual bleeding or spotting		
No	3.46 (0.79–15.12)	0.099
Yes	ref	
Menstrual bleeding interference with daily life activities		
Not at all	ref	0.052
Mild	4.16 (1.38–12.56)	
Moderate	1.37 (0.43–4.39)	
Change in the menstrual bleeding’s interference with daily life activities measured from basal visit to final visit		
Not at all/mild/moderate	ref	**0.0063**
Intense	3.89 (1.47–10.33)	
Change in the satisfaction with the menstrual bleeding pattern measured from basal visit to final visit		
Improvement	ref	0.1283
No change	1.459 (0.130–16.375)	
Worsening	7.417 (0.998–55.121)	

*p* Values in bold indicate statistical significance.

^a^ Wald test from logistic regression.

In addition, a linear correlation was performed between two variables, and only the intensity of menstrual bleeding exhibited statistically significant associations with discontinuation. Interestingly, in women with a 12-month follow-up, the change in the pain associated with menstrual bleeding measured from the basal visit to the final visit exhibited statistically significant associations with the discontinuation. The group of women who discontinued the use of the LNG-IUS had, on average, higher pain levels compared with those who did not discontinue (50.5 ± SD 13.96 vs. 12.01 ± SD 27.7; *p* = 0.0086) (Supplementary Table S1).

### Satisfaction rate

The low discontinuation rates observed after 1 year of use led us to investigate the impact on the devices satisfaction rates. When evaluating the association between satisfaction with the menstrual bleeding pattern and preinsertion information received in the whole sample, women who received a lot or quite a lot of information showed higher rates of satisfaction (48.3% and 40.0%, respectively) to those who received relatively little or very little information (3.3% and 0%, respectively) (Supplementary Table S2). Moreover, women whose menstrual bleeding pattern did not interfere with their daily life activities were also the most satisfied with the device (*p* < 0.0001) (Supplementary Table S3). Finally, 91.04% of women stated at the final visit that they would recommend low-dose LNG-IUS to peers. Those most satisfied with their menstrual pattern were also more likely to recommend it (data not shown).

## Discussion

### Findings and interpretation

This study reports the 12-month continuation rate of low-dose LNG-IUS among Spanish users. Among basal factors potentially impacting the continuation rate, educational level, previous pregnancies, and menstrual pain were identified. Moreover, associations between discontinuation rate and menstrual bleeding’s interference with daily life activities were also observed.

Although LARCs are effective, safe, and highly recommended contraceptive methods,^1,2,11^ their use is not widespread in Spain. According to “Survey on Contraception in Spain-2022,” only 8.4% of women aged 14–49 are using LARCs (either 3.8% LNG-IUS, 2.9% cooper IUD, or 1.7% implants).^5^ These figures point out the presence of barriers to using these methods, regardless of their recognized efficacy. In fact, its use has increased extremely little in the last years: only 1.3% from 2014 to 2022.^5,12^

The availability of new LNG-IUS in Spain has introduced the need of assess factors that may influence the effectiveness of these methods in real-life, beyond contraceptive efficacy. Understanding reasons underlying discontinuation of the LNG-IUS in the real world due to its vital role in preventing unintended pregnancies. In this study, low-dose LNG-IUS discontinuation due to undesirable changes in menstrual bleeding or adverse events was low. Overall, 9% of participants discontinued the low-dose LNG-IUS, and among them, 2.4% discontinued due to unacceptable menstrual bleeding patterns during the 12-month observation period. This observation is in line with figures reported from phase II and III studies with 19.5 mg LNG-IUS, where 2.8% of participants discontinued because of bleeding problems (including absence of bleeding) at 12 months.^8^ In real-world studies, total discontinuation rates between 9.3% and 45% have been reported.^13,14^ Regarding discontinuation due to bleeding problems at 12 months, previous studies have reported rates of 1.8%, in line with the present results.^15^

### Results in the context of what is known

When considering baseline characteristics’ impact on the continuation rate, a significant relationship between the educational level or previous pregnancies of study participants and discontinuation rates was observed. It may be that it is not simply educational level, but health literacy and the ability to understand provided information that is key to satisfaction with IUS. Moreover, although previous studies have reported no correlation between parity and low-dose LNG-IUS discontinuation,^16^ Simmons et al. identified as a factor associated with discontinuation having any future pregnancy plans, even years out. However, in the present study, the desire to have more children despite previous pregnancies was not associated with discontinuation. Simmons et al. also reported that participants with some college education were less likely to report discontinuation (incidence rate ratio, 0.73; 95% CI: 0.57, 0.94).^17^ It is important to highlight that up to 85% of LNG-IUS users included in the present study reported high school/tertiary or university studies. Finally, baseline menstrual pain also showed a significant association with discontinuation, with higher pain levels reported by women who discontinued LNG-IUS usage. Whether or not these three factors influenced the low-dose LNG-IUS discontinuation rate, these results could be helpful to women when considering method choice prior to placement. Understanding factors that contribute to a woman’s decision to continue or discontinue could help women in their choice of contraception and type of IUS.^17^

### Clinical implications

Although discontinuation for an unacceptable menstrual bleeding pattern constituted less than a third of cases of discontinuation in the present study, this is commonly reported as a reason for discontinuation. Depending on the cultural context, amenorrhea is perceived as indicative of illness, infertility, or even death.^10^ Apart from beliefs and misconceptions, menstrual bleeding changes due to contraception use may have a notable impact on daily life.^10^ In fact, in the present study, LNG-IUS discontinuation was associated with reporting intense menstrual bleeding’s interference with daily life activities measured from basal visit to final visit. Therefore, it is of paramount importance to adequately educate women to fully understand, anticipate, or manage the potential changes in their menstrual bleeding pattern due to the LNG-IUS use. Especially in young women, who want to understand not only benefits but also side effects of their contraceptive method of choice besides pregnancy prevention.^18^ Thus, HCPs should be especially aware of the variables impacting long-term women’s satisfaction with the LNG-IUS in order to provide better advice.^10^ HCP counseling has to be comprehensive enough to improve women’s satisfaction with the IUD and its continuation. According to the TANCO study, HCPs tend to underestimate women’s interest in receiving information on contraception in general and, more specifically, LARC methods.^19^

Given that the mean time to discontinuation in the present study was 213 days (SD ± 134.07 days), that is, approximately after 7 months, with 380 days (SD ± days) for 13.5 mg LNG-IUS users and 206.32 days (SD ± 132.35 days) for 19.5 mg LNG-IUS users, it states that discontinuation does not occur in the first months after insertion (data not shown). Importantly, it has been reported that discontinuation due to adverse events, device expulsion, or pregnancy tends to be even lower in later years (3–5) when compared with earlier times (1–2 years).^20^ Furthermore, it is also reported^21^ that during a 5-year period, 22.6% discontinued using LNG-IUS due to treatment-emergent adverse events, including 163 women who discontinued because of desire for pregnancy.^21^

In this study, women who received quite a lot/a lot of information showed higher rates of satisfaction (88%) with the device, although no correlations with discontinuation were observed. Given that experience and satisfaction with the device play an important role in continuation, potential improvements in counseling and pain management could increase satisfaction and continuity with the low-dose LNG-IUS.

### Future directions

Future research should include larger studies with balanced groups that simultaneously assess discontinuation rates and potential impact factors. To clarify the real impact of these factors, extended follow-up studies up to 3–5 years (estimated duration time of LNG-IUS) and analysis of correlation between discontinuation rates and other parameters such as adverse events, satisfaction, and bleeding patterns would be also interesting.

### Strengths and limitations

This study represents a novel contribution to the literature as it investigates basal factors impacting low-dose LNG-IUS discontinuation, which has not been extensively explored in previous research. These findings can significantly enhance method continuation, contraceptive counseling, and user satisfaction. In the present study, women attending gynecological consultations who had chosen an LNG-IUS as a contraceptive method for the first time at the baseline visit were consecutively recruited, which prevents intentional selection of women and makes it possible to incorporate a random effect (consultation attendance order). The study collected data over 12 months, providing valuable insights into the long-term continuation rates and factors influencing LNG-IUS discontinuation. A detailed CRF was used to collect demographic and clinical data, ensuring a comprehensive assessment of factors influencing LNG-IUS discontinuation rates. Moreover, collaboration with multiple health care centers across Spain enhances the external validity of the study findings and increases the generalizability of the results to diverse clinical settings and patient populations. The study ensured geographical diversity by including patients from various regions across Spain, reducing the risk of geographical sample bias and enhancing the representativeness of the study population.

Limitations of this study include, firstly, inherent limitations in the observational design, which prevent the inference of causal relationships. There may be unaccounted confounding variables or statistical analyses that could potentially impact the results. Secondly, the sample distribution was unbalanced, and results may be biased because of the number of patients included in each group. Indeed, the 19.5 mg LNG-IUS group included almost 10-fold patients than 13.5 mg LNG-IUS group. It may be explained by the longer duration of 19.5 mg LNG-IUS, which is recommended for 5 years use,^22^ versus 3 years for 13.5 mg LNG-IUS.^23^ The longer duration of LNG-IUS 19.5 mg (5 years) as compared with LNG-IUS 13.5 mg can explain the imbalance of the sample distribution among the available low-dose LNG-IUS.^15^ Another reason arises from the fact that 19.5 mg LNG-IUS is offered free of charge for women with a history of voluntary termination of pregnancy in many Spanish autonomous communities. Lastly, another limitation is the lack of information on 26 women (8% of the enrolled sample) whose continuity in the use of the LNG-IUS is unknown. This uncertainty may introduce bias in the analysis. If all these women had discontinued the use of the LNG-IUS, the study’s results and conclusions could differ. This underscores the need to interpret the findings cautiously and the importance of minimizing loss to follow-up in future studies.

While the study collected data over 12-months, longer follow-up durations could provide additional insights into the factors influencing LNG-IUS continuation rates over time. The study relied on self-reported data for certain variables, such as menstrual bleeding patterns and satisfaction with the device. This introduces the possibility of recall bias, which may affect the accuracy of the reported outcomes. A considerable number of participants did not complete the follow-up visits, resulting in missing data and potential bias in the analysis of discontinuation rates and associated factors. This loss to follow-up could impact the reliability and validity of the study findings. The study was conducted exclusively among Spanish women, so the results may not be directly applicable to other populations or cultural settings. The findings cannot be extrapolated to other contraceptive methods, except for low-dose LNG-IUS.

## Conclusions

This study highlights the need to disentangle factors associated with low-dose LNG-IUS discontinuation due to changes in bleeding patterns in routine clinical practice. By understanding and addressing these concerns, health care providers could improve contraceptive counseling, reduce discontinuation rates, and enhance women’s satisfaction with their chosen contraceptive method. The findings also shed light on the significance of preinsertion counseling. In addition, the study underscores the importance of considering individual pain levels and menstrual bleeding’s interference with daily life activities when recommending LNG-IUS to women, as these factors can influence the overall satisfaction and continuation of the contraceptive method.

## Data Availability

Bayer submitted the information of this observational study to the publicly funded website www.ClinicalTrials.gov. under the NCT number 04785950. Availability of the data underlying this publication will be determined according to Bayer’s commitment to the EFPIA/PhRMA “Principles for responsible clinical trial data sharing.” This pertains to the scope, timepoint, and process of data access. As such, Bayer commits to sharing upon request from qualified scientific and medical researchers patient-level clinical trial data, study-level clinical trial data, and protocols from clinical trials in patients for medicines and indications approved in the United States (US) and European Union (EU) as necessary for conducting legitimate research. This applies to data on new medicines and indications that have been approved by the EU and US regulatory agencies on or after January 01, 2014. Interested researchers can use www.vivli.org to request access to anonymized patient-level data and supporting documents from clinical studies to conduct further research that can help advance medical science or improve patient care. Information on the Bayer criteria for listing studies and other relevant information is provided in the member section of the portal. Data access will be granted to anonymized patient-level data, protocols, and clinical study reports after approval by an independent scientific review panel. Bayer is not involved in the decisions made by the independent review panel. Bayer will take all necessary measures to ensure that patient privacy is safeguarded.

## Abbreviations Used

AEMPS: Agencia Española de Medicamentos y Productos Sanitarios
CI: Confidence Interval
FAS: Full Analysis Set
HCP: Health Care Professionals
IRR: Incidence Rate Ratio
IUD: Intrauterine Devices
LARC: Long-Acting Reversible Contraceptives
LNG-IUS: Low-Dose Levonorgestrel-Releasing Intrauterine Systems
OR: Odds Ratio
SD: Standard Deviation
