# Supplementary material for: Understanding Discontinuation Rates and Acceptance of the Low-Dose Levonorgestrel Intrauterine System in Spain: A Comprehensive Analysis of Bleeding Patterns and Influencing Factors
Source: Womens Health Rep (New Rochelle). 2025 Mar 5;6(1):209–20. doi: 10.1089/whr.2024.0113 (PMC11931107; doi:10.1089/whr.2024.0113)
Supplement: Supplementary Table S1 [file whr.2024.0113_supplementary_table_s1.docx]

**Supplementary** **Table 1.** Association between changes at 12 months and low-dose LNG-IUS discontinuation or continuation

|  | **Women who discontinued the use of low-dose LNG-IUS** | **Women who continued with the low-dose LNG-IUS** | **Total** | **p-value*** |
| --- | --- | --- | --- | --- |
| **Change in the pain associated to the intensity of menstrual bleeding (T0-T12),** (mm) mean (SD) | 50.5 (13.96) | 12.01 (27.7) | 13.13 (28.12) | **0.0086** |

*Mann–Whitney–Wilcoxon test

p-values in bold indicate statistical significance.
