# Supplementary material for: Understanding Discontinuation Rates and Acceptance of the Low-Dose Levonorgestrel Intrauterine System in Spain: A Comprehensive Analysis of Bleeding Patterns and Influencing Factors
Source: Womens Health Rep (New Rochelle). 2025 Mar 5;6(1):209–20. doi: 10.1089/whr.2024.0113 (PMC11931107; doi:10.1089/whr.2024.0113)
Supplement: Supplementary Table S2 [file whr.2024.0113_supplementary_table_s2.docx]

**Supplementary** **Table 2.** Women’s perception of the amount of information received during pre-insertion counseling about possible changes in menstrual bleeding pattern after low-dose LNG-IUS insertion (T12)

|  | **Satisfied/**  **Very satisfied**  n= 60 (22.39%) | **Neither satisfied nor dissatisfied**  n= 204 (76.12%) | **Dissatisfied/Very Dissatisfied**  n= 4 (1.49%) | **Total**  n= 268 (100.0%) | **p-value*** |
| --- | --- | --- | --- | --- | --- |
| **Amount of information received** |  |  |  |  | **0.0158** |
| **Quite a** lot | 29 (48.3%) | 72 (35.3%) | 0 (0.0%) | 101 (37.7%) |  |
| **A** lot | 24 (40.0%) | 109 (53.4%) | 1 (25.0%) | 134 (50.0%) |  |
| A moderate amount | 5 (8.3%) | 18 (8.8%) | 2 (50.0%) | 25 (9.3%) |  |
| Relatively little | 2 (3.3%) | 4 (2.0%) | 1 (25.0%) | 7 (2.6%) |  |
| Very little | 0 (0.0%) | 1 (0.5%) | 0 (0.0%) | 1 (0.4%) |  |

*Fisher's Exact Test. p-values in bold indicate statistical significance.
