# Supplementary material for: Understanding Discontinuation Rates and Acceptance of the Low-Dose Levonorgestrel Intrauterine System in Spain: A Comprehensive Analysis of Bleeding Patterns and Influencing Factors
Source: Womens Health Rep (New Rochelle). 2025 Mar 5;6(1):209–20. doi: 10.1089/whr.2024.0113 (PMC11931107; doi:10.1089/whr.2024.0113)
Supplement: Supplementary Table S3 [file whr.2024.0113_supplementary_table_s3.docx]

Supplementary Table 3. Association between change in the presence of intermenstrual bleeding or spotting measured from basal visit to final visit and users’ satisfaction with the menstrual bleeding pattern

|  | **Satisfied/**  **Very satisfied**  **N=60 (22.39%)** | **Neither satisfied nor dissatisfied**  N=**204 (76.12%)** | **Dissatisfied/Very Dissatisfied**  N=**4 (1.49%)** | **Total**  N=**268 (100.0%)** | **p-value*** |
| --- | --- | --- | --- | --- | --- |
| Change in the presence of intermenstrual bleeding or spotting measured from basal visit to final visit (T0-T12) |  |  |  |  | **<0.0001** |
| Absence of menstrual bleeding (basal and/or final visit) | 18 (30.0%) | 75 (36.8%) | 1 (25.0%) | 94 (35.1%) |  |
| Improvement | 5 (8.3%) | 62 (30.4%) | 2 (50.0%) | 69 (25.7%) |  |
| No change | 33 (55.0%) | 52 (25.5%) | 1 (25.0%) | 86 (32.1%) |  |
| Worsening | 4 (6.7%) | 15 (7.4%) | 0 (0.0%) | 19 (7.1%) |  |

*Fisher's Exact Test. p-values in bold indicate statistical significance.
